# A DNA Contact Map for the Mouse *Runx1* Gene Identifies Novel Haematopoietic Enhancers

**DOI:** 10.1038/s41598-017-13748-8

**Published:** 2017-10-17

**Authors:** Judith Marsman, Amarni Thomas, Motomi Osato, Justin M. O’Sullivan, Julia A. Horsfield

**Affiliations:** 1 Department of Pathology, Dunedin School of Medicine, University of Otago, Dunedin, New Zealand; 2 Cancer Science Institute, National University of Singapore, Singapore, 117599 Singapore; 3 International Research Center for Medical Sciences, Kumamoto University, Kumamoto, Japan; 4 Liggins Institute, The University of Auckland, Private Bag, 92019 Auckland, New Zealand; 5 Maurice Wilkins Centre for Molecular Biodiscovery, The University of Auckland, Private Bag, 92019 Auckland, New Zealand

## Abstract

The transcription factor Runx1 is essential for definitive haematopoiesis, and the *RUNX1* gene is frequently translocated or mutated in leukaemia. *Runx1* is transcribed from two promoters, P1 and P2, to give rise to different protein isoforms. Although the expression of Runx1 must be tightly regulated for normal blood development, the mechanisms that regulate *Runx1* isoform expression during haematopoiesis remain poorly understood. Gene regulatory elements located in non-coding DNA are likely to be important for *Runx1* transcription. Here we use circular chromosome conformation capture sequencing to identify DNA interactions with the P1 and P2 promoters of *Runx1*, and the previously identified +24 enhancer, in the mouse multipotent haematopoietic progenitor cell line HPC-7. The active promoter, P1, interacts with nine non-coding regions that are occupied by transcription factors within a 1 Mb topologically associated domain. Eight of nine regions function as blood-specific enhancers in zebrafish, of which two were previously shown to harbour blood-specific enhancer activity in mice. Interestingly, the +24 enhancer interacted with multiple distant regions on chromosome 16, suggesting it may regulate the expression of additional genes. The *Runx1* DNA contact map identifies connections with multiple novel and known haematopoietic enhancers that are likely to be involved in regulating *Runx1* expression in haematopoietic progenitor cells.

## Introduction

Runx1 is a key regulator of haematopoietic development. Deletion of *Runx1* in mouse embryos is lethal in embryonic stage (E) 12.5 due to the complete absence of definitive blood cell progenitors accompanied by extensive haemorrhaging^1,2^. Runx1 is crucial for haematopoietic stem cell (HSC) emergence and maintenance during development^3^, since conditional ablation of *Runx1* in adult mice results in HSC exhaustion^4^. In acute myeloid leukaemia (AML) and myelodysplastic syndrome, RUNX1 function is frequently altered through mutations or translocations^5^, resulting in dysregulation of its target genes. While mutations directly affecting the RUNX1 protein are common in leukaemia, mutations in regulatory elements that affect *RUNX1* expression remain enigmatic. As yet unidentified mutations in regulatory elements, such as enhancers, could alter *Runx1* expression, resulting in abnormal haematopoiesis.

*Runx1* is transcribed from two promoters, P1 and P2, to give rise to different protein isoforms^6^. Expression of these isoforms is tightly controlled during haematopoiesis. At the onset of mouse haematopoiesis (E7.5), preceding the generation of HSCs, expression of the P2 isoform(s) is predominant^7,8^. P1 is expressed soon after P2, and its expression is synchronised with the generation of HSCs^7,9^. P1 expression is predominant in the mouse fetal liver, the main site of definitive haematopoietic stem/progenitor cell (HSPC) development from E12.5 onward^9^.

Regulatory elements, such as enhancers, can control the expression of genes via long-range chromatin interactions^10^. One previously identified *Runx1* enhancer is located 24 kb downstream of the P1 transcriptional start site^11,12^. The +24 enhancer (also known as the +23^13^, or the +23.5^12^ enhancer) is active in HSCs that express *Runx1* during mouse embryogenesis^11–13^. The human equivalent of the +24 enhancer (+32 kb downstream of P1) directly contacts the promoters of *RUNX1* in leukaemia cell lines^14^. In addition to the +24 enhancer, putative regulatory elements for *RUNX1* have been identified upstream of *RUNX1*-P1 and between P1 and P2; however, whether they directly contact the *RUNX1* promoters has not been investigated^15,16^.

Here we used circular chromosome conformation capture sequencing (4C-seq) to identify regulatory elements that interact with an active *Runx1* P1 promoter, versus an inactive P2 promoter. While Hi-C provides genome-wide contact profiles, and Capture Hi-C enriches for interactions with preselected genomic features (usually promoters), 4C-seq can generate very high resolution contact profiles from ‘baits’ of particular interest^17^. Therefore, 4C-seq can yield richer information about a selected genomic region than Hi-C or Capture Hi-C. HPC-7 is a well characterised mouse HSPC line with genomic annotations, including transcription factor (TF) binding, histone modifications and chromatin accessibility^18–20^. 4C-seq in HPC-7 cells identified nine haematopoietic enhancers that interact with the P1 promoter and +24 enhancer, and that are occupied by haematopoietic TFs. Four of these were previously identified, three of which were functionally tested and two out of the three showed activity during mouse haematopoiesis^15^. Here we assessed the activity of all nine enhancers in zebrafish, and show that eight are active in zebrafish haematopoiesis. Further, the +24 enhancer was highly interactive both within a topologically associated domain (TAD) harbouring *Runx1*, as well as with loci outside the TAD. Collectively, our results point to the formation of a local ‘active chromatin hub’ controlling *Runx1* expression in haematopoietic cells.

## Results

**Figure 1:**
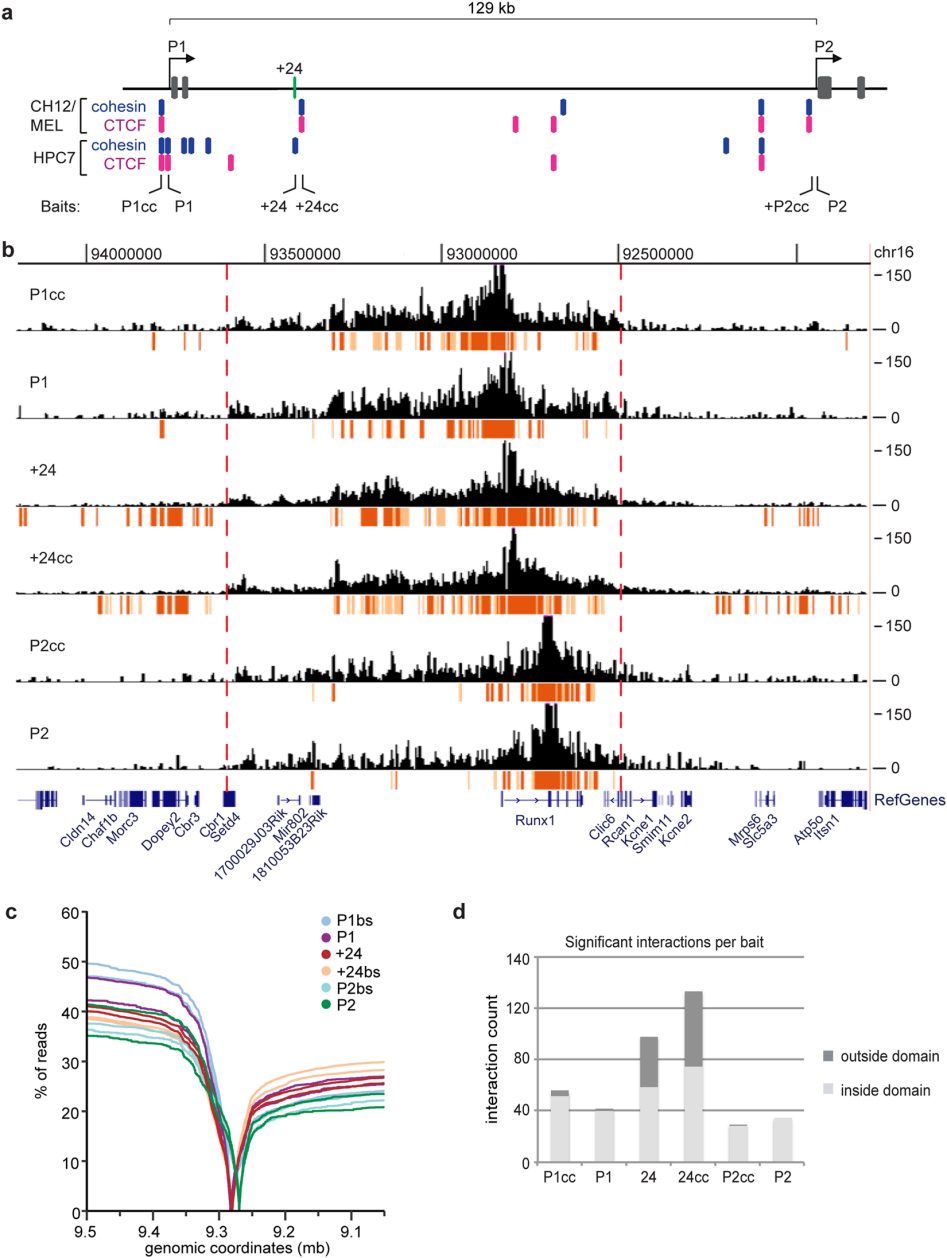
*Runx1* interaction profile reflects the activity of the P1 and P2 promoters and the +24 enhancer. (**a**) Location of 4C baits at the mouse *Runx1* gene (mm10). Baits are P1cc, P1, +24, +24cc, P2cc and P2; ‘cc’: cohesin and CTCF binding site. ChIP-seq data of Rad21 (blue) and CTCF (pink) in MEL and CH12 cells were obtained from ENCODE^51^ and in HPC-7 cells from^18,19^. Exons are in grey and the +24 enhancer is in green. (**b**) 4C-seq profile (read per million normalised running mean of nine successive *Dpn*II digestion fragments) for one replicate per bait with significant interactions (the top fifth percentile of interactions with a FDR of <0.01) that overlap in both replicates shown in a ~2.5 Mb region surrounding the *Runx1* gene. The highest significant interactions (see Methods) are in red and other significant interactions in orange. Red dotted lines indicate the location of estimated domain boundaries. (**c**) Read distribution for both replicates of each bait. Line graphs represent the cumulative percentage of reads on chromosome 16 (y-axis) versus the distance from the bait (x-axis), shown in genomic coordinates on chromosome 16 (genome version mm10). The 0-value of each line indicates the location of each bait. (**d**) The number of significant interactions that overlap in both replicates inside (light grey) and outside (dark grey) the domain for each bait.

We first confirmed that P1 is actively expressed in HPC-7 cells, while P2 is silent (Supplementary Fig. S1). 4C-seq in HPC-7 cells using the P1 and P2 promoters and the +24 enhancer as ‘baits’ identified genomic interactions at *Runx1* (Fig. 1a). Bait locations were designed taking into account cohesin and CTCF binding sites near both promoters and the +24 enhancer. 4C baits were designed to regions of interest (P1, +24, P2), allowing for comparison of interactions between the active P1 promoter and inactive P2 promoter, with secondary baits located at nearby cohesin/CTCF (cc) binding sites (P1cc, +24cc, P2cc) (Fig. 1a).

### 4C-seq in HPC-7 cells confirms the presence of a 1.1 Mb domain harbouring *Runx1*

For each bait, two replicate 4C-seq libraries and one control library were sequenced. Reads were predominantly located within a 1.1 Mb region surrounding the *Runx1* gene (Fig. 1b,c), representing a TAD harbouring *Runx1*. From visual inspection of the contact profile (Fig. 1b,c), domain boundaries appear to be present at the *Cbr1*/*Setd4* genes upstream of *Runx1*, and *Clic6* downstream (Fig. 1b). A comparison with existing Hi-C data from mouse embryonic stem cells, mouse CH12 (erythroleukaemia) cells, human GM12878 (lymphoblastoid), human K562 (myeloid leukaemia) and human IMR90 (foetal lung) cells revealed that TAD boundaries are conserved, and that our 4C data is consistent with existing Hi-C data (Supplementary Fig. S2). Most P1 and +24 interactions take place upstream of the *Runx1* gene (Fig. 1b,c). In contrast, there are fewer upstream interactions from P2, while downstream interactions are retained (Fig. 1b,c). There are no other coding genes within the *Runx1* domain; however, three inactive non-coding genes^19^, *Mir802* and the long non-coding RNAs *1810053B23Rik* and *1700029J03Rik*, are located near the upstream border of the TAD.

Interactions from ‘cc’ baits (cohesin/CTCF binding sites) were similar to their nearby corresponding baits at P1, +24 and P2 (Fig. 1b). We investigated whether significant interactions for the ‘cc’ baits have more overlap with other cohesin or CTCF binding sites than for the non ‘cc’ baits, but did not find any difference (data not shown). The ‘cc’ baits may be too close to the corresponding P1, P2 and +24 baits to resolve unique interactions, although they are separated by at least one restriction fragment.

### Chromatin interactions anchored by the *Runx1* P1 and P2 promoters and +24 enhancer

**Figure 2:**
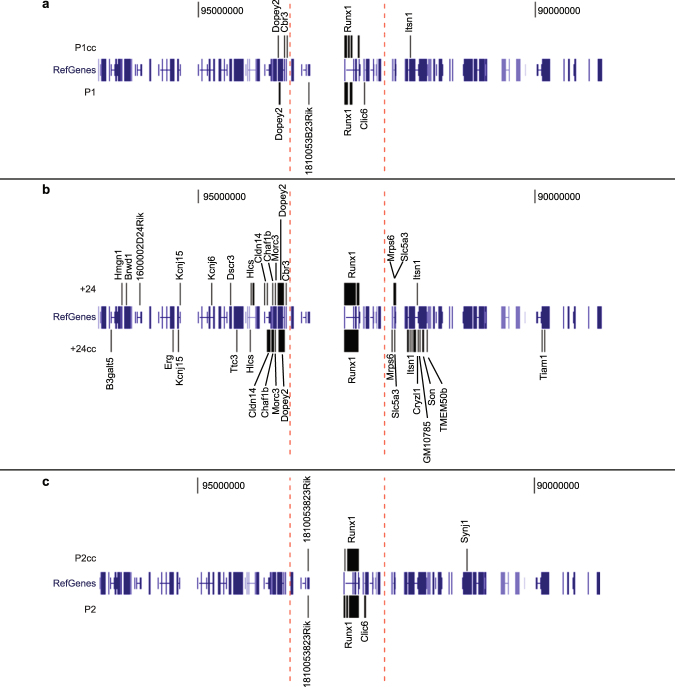
Interactions of the *Runx1* locus with other genes. Significant interactions that overlap with gene bodies or gene promoters (starting from 2 kb upstream of the gene transcriptional start site) are shown for the P1cc/P1 (**a**), +24/ +24cc (**b**) and P2cc/P2 baits (**c**) (assembly mm10).

Most of the significant interactions for P1, P2 and the +24 enhancer occur within the 1.1 Mb domain (Fig. 1b). There are ~40–50 significant interactions for P1cc/P1 baits, ~60–75 for +24/+24cc baits, and ~30 for P2/P2cc baits (Fig. 1d). Strikingly, the +24 enhancer in particular forms many significant interactions outside the *Runx1* domain (Fig. 1b,d). Many of these long-range interactions are with other genes or gene promoters on mouse chromosome 16 (Fig. 2). Expression levels of these genes in human tissues according to Genotype-Tissue Expression (GTEx, https://www.gtexportal.org/home/)^21^ did not reveal obvious tissue-specific expression patterns. However, among the genes contacted by +24 are *Erg*, a haematopoietic TF, and *Tiam1* (T-cell lymphoma invasion and metastasis 1), involved in cell adhesion and cell migration. These distant connections suggest that +24 may also regulate other haematopoietic genes in adjacent domains.

### Identification of haematopoietic enhancers

**Figure 3:**
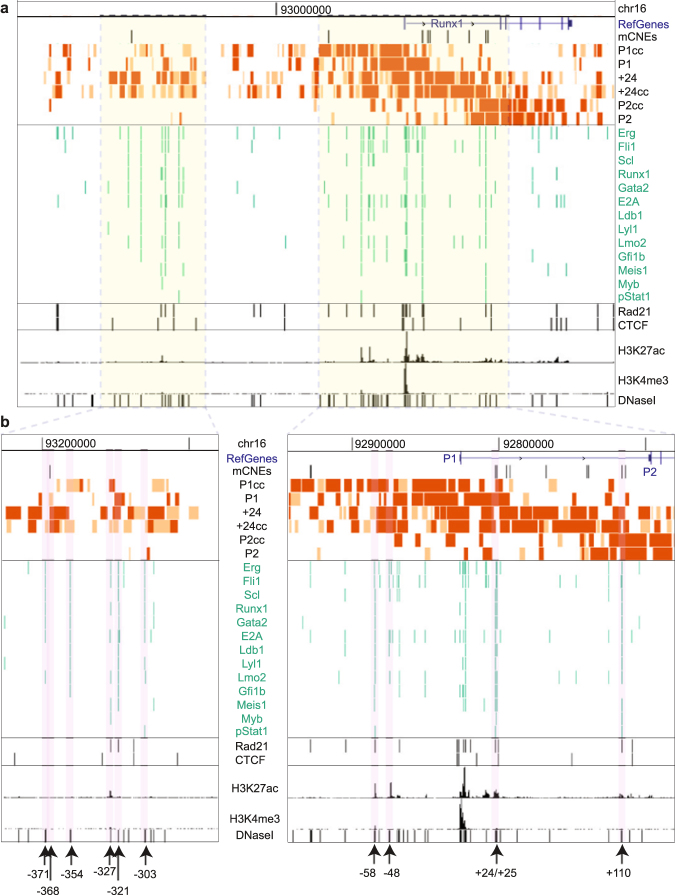
The active P1 promoter and +24 enhancer interact with clusters of haematopoietic transcription factor binding sites. Significant interactions within the domain for each bait are shown together with the reference genes (mm9), CNEs^11^, TF binding sites (green), Rad21 and CTCF binding sites (black), H3K27ac, H3K4me3 and DNaseI hypersensitivity sites in HPC-7 cells^18–20^. A 3 Mb region (**a**) and two zoomed-in views (**b**) are shown. Locations of TF binding clusters containing at least six TF binding sites at the same location, and a significant interaction directly at or within 2 kb of each cluster, are indicated by arrows and named according to their distance from the P1 transcriptional start site. See also Supplementary Fig. S3.

We hypothesised that DNA connections formed from the active P1 promoter and the +24 enhancer may correspond to haematopoietic enhancers that regulate *Runx1* expression. To identify putative enhancers, we aligned significantly interacting sites with the occupancy of thirteen TFs involved in haematopoietic progenitor cell production; enhancer histone modifications and DNase I hypersensitivity sites^18–20^; and conserved non-coding elements (CNE), which were identified based on comparative genomics alignment and retroviral integration site mapping to indicate potential DNaseI hypersensitive sites^11^. We note that the +24 enhancer binds all thirteen haematopoietic progenitor TFs in HPC-7 cells (Fig. 3).

We selected putative enhancers based on the binding of at least six TFs involved in haematopoietic progenitor cell development (Fig. 3), and the presence of a significant interaction directly at, or within 2 kb, of the TF binding cluster. Based on these criteria, we found eight other potential enhancers within the *Runx1* domain that form connections to the baits. These enhancers were named according to their distance from the P1 transcriptional start site: −371, −354, −327, −321, −303, −58, −48, and +110 (Fig. 3b). An additional interacting region at −368, a CNE, was located adjacent to a cluster of haematopoietic TFs (Fig. 3b). The +24 and +110 are also CNEs^11^. The putative enhancers either form distinct blocks upstream of *Runx1*-P1 (from −371 to −303 and −48 to −58), or fall between the P1 and P2 promoters (+24 and +110). Four out of nine of the identified putative enhancers (−327, −321, −58 and +110) were identified previously based on the binding of at least three blood TFs and the presence of a H3K27ac peak (denoting active chromatin) from a subset of the same HPC-7 datasets that we have used here^15^.

**Figure 4:**
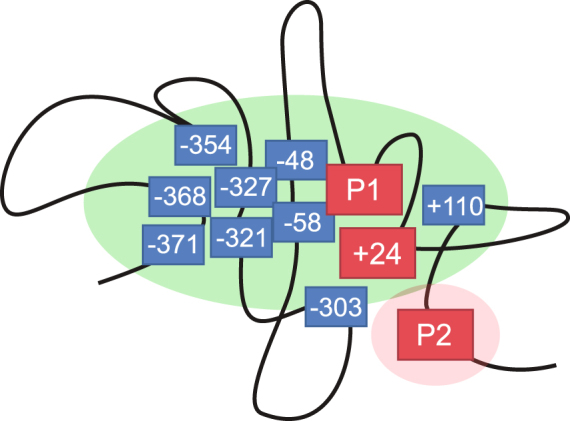
Model of chromatin interactions based on *Runx1* 4C-seq. 4C-seq baits are shown in red and identified interacting enhancers in blue. P1 is active, and P2 is inactive. P1 interacts with −371, −368, −354, −327, −321, −58, −48, +24 and +110; +24 interacts with −371, −368, −354, −327, −321, −303, −58, −48, +110 and P2; and P2 interacts with −303 and +24.

Eight out of nine putative enhancers (−371, −368, −354, −327, −321, −58, −48, and +110) form long-range interactions with the P1 promoter, the +24 enhancer, or both (Fig. 3b and Supplementary Table S1). The exception was −303, which does not interact with P1, but instead with the +24 and the P2 promoter. The +24 enhancer interacts promiscuously within the whole domain. Filtering at maximum stringency (see Methods) showed that the +24 enhancer connects to all putative enhancers and both *Runx1* promoters (Fig. 3b). In contrast, the inactive P2 promoter connects only to −303 and +24 (Fig. 3b and Supplementary Table S1). A specific interaction between +110 and P2 could not be resolved owing to a contiguous block of significant interactions throughout the short region between +110 and P2. A model of *Runx1* interactions based on the 4C-seq results is shown in Fig. 4.

### Long range chromatin interactions at haematopoietic enhancers

Long-range chromatin interactions can be mediated by cohesin and CTCF^22^, and cohesin is involved in transcription regulation at active genes^23^. We found that four of the nine enhancer loci (in addition to the +24 enhancer) coincide with Rad21 (cohesin) binding in the absence of CTCF (Fig. 3b and Supplementary Table S1). This is consistent with the idea that cohesin (but not necessarily CTCF) mediates local DNA-DNA interactions within TADs^22^. All Rad21 binding sites interacted with at least one ‘cc’ bait, therefore cohesin could mediate at least a subset of enhancer-promoter communication events in HPC-7 cells.

We compared the 4C interactions identified with recently published Capture Hi-C data in HPC-7 cells^18^ (Supplementary Fig. S3). Capture Hi-C data was only available for interactions anchored at P1, and has a lower coverage and resolution than our 4C-seq study (an average of ~18,000 reads per promoter for Capture Hi-C with a 6-cutter, and over 1 million reads per bait for 4C-seq with a 4-cutter). The Capture Hi-C study in HPC-7 cells identified 15 P1-interacting regions that were reproduced in our study (Supplementary Fig. S3). All of these are upstream of *Runx1*-P1, and most are within the −368 to −303 enhancer cluster (Supplementary Fig. S3). Therefore, our study provides additional *Runx1*-anchored interactions that were not previously described as connected to *Runx1* promoters (enhancers −371, −48, −58 and +110).

### *In vivo* characterization of haematopoietic enhancers

**Figure 5:**
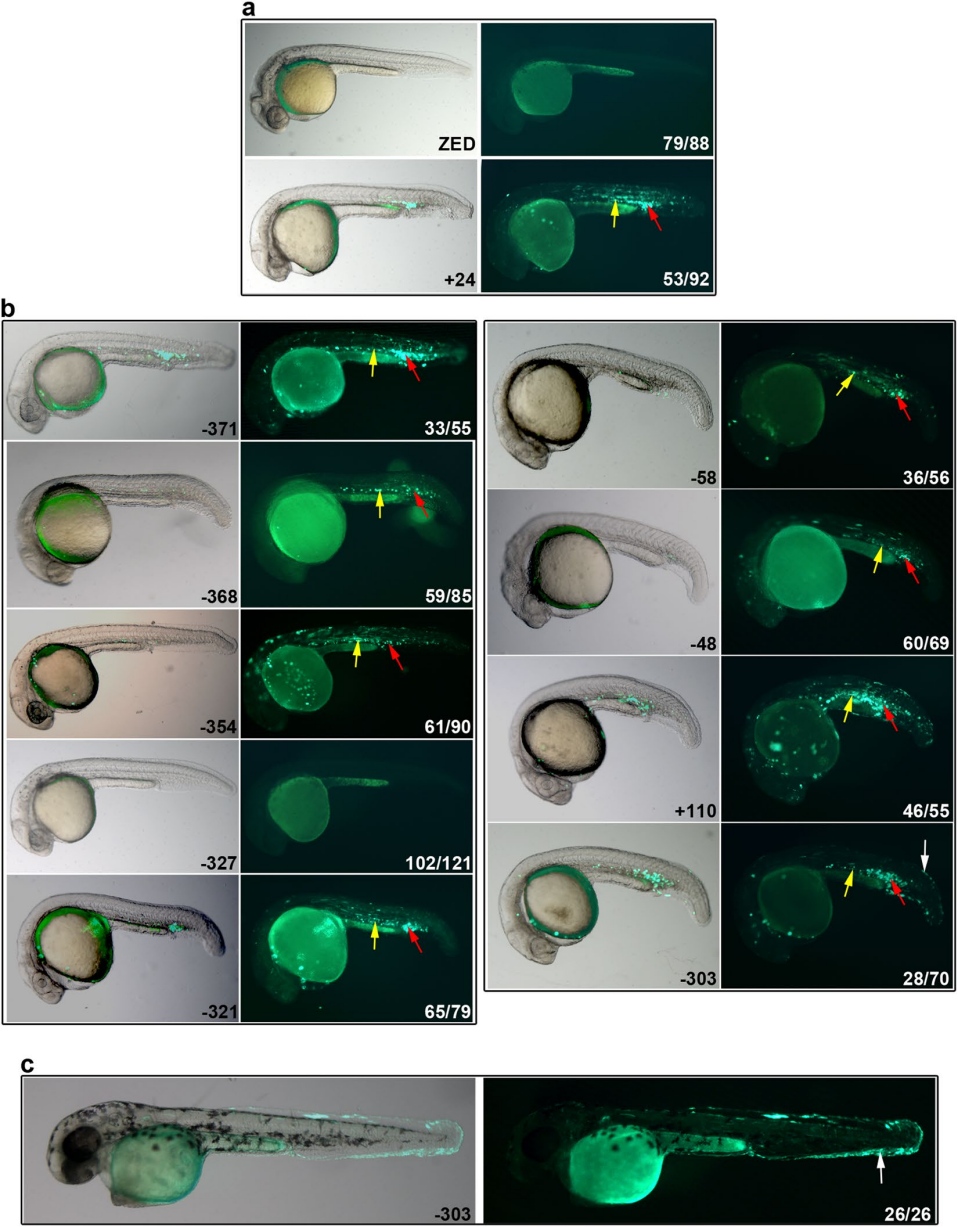
Putative mouse enhancers are active in haematopoietic regions in zebrafish. Whole mount representative lateral images of zebrafish embryos (20–24 hpf [**a**,**b**] and ~48 hpf [**c**]) that were injected with enhancer-GFP plasmids at the one-cell stage; left hand panels are merges with bright field, right hand panels are GFP fluorescence. (**a**) Negative control ZED plasmid shows zero (image) or non-specific fluorescence, while the positive control (+24) shows GFP expression in haematopoietic cells of the posterior blood island (red arrows) and intermediate cell mass (yellow arrows), which  represent primitive blood cells. (**b**) Enhancer activity of +110, −48, −58, −303, −321, −327, −354, −368, −371. GFP expression was observed in primitive haematopoietic cells of the posterior blood island (red arrows) and intermediate cell mass (yellow arrows). **(c)** The −303 enhancer also expressed GFP in keratinocytes (white arrows in **b,c**). The numbers in the right hand panels represent the number exhibiting the representative phenotype out of the total number of fluorescent embryos analysed. See also Supplementary Fig. S4.

Enhancer regions interacting with *Runx1* recruit haematopoietic TFs in HPC-7 cells, therefore we determined if these regions act as enhancers *in vivo*. Each of the putative enhancers was tested for the ability to drive tissue-specific GFP expression in zebrafish embryos. Eight out of nine drove GFP expression specifically in the intermediate cell mass and posterior blood island, which are sites of haematopoietic progenitor cell production at 20–24 hours post-fertilisation (hpf)^24^ (Fig. 5a,b and Supplementary Fig. S4). Two of these (−58 and +110) were previously shown to be active during mouse haematopoiesis^15^. The −303 enhancer also expressed GFP in keratinocytes, particularly after 24 hpf (Figs 5c, S4). Interestingly, −303 was the only enhancer identified that interacted with the P2 promoter of *Runx1*, rather than P1. Despite the occupancy of multiple haematopoietic TFs in HPC-7 cells, and the presence of similar TF binding motifs compared to the other enhancers (Fig. 3 and Supplementary Tables 1 and 2, we did not observe enhancer activity for −327, consistent with a previous study in mice^15^.

In summary, we have assigned *in vivo* function to multiple putative enhancers upstream of *Runx1* that were previously identified *in silico* ^18^. These regions not only drive haematopoietic expression, but also physically connect with the *Runx1*-P1 promoter, lending confidence to the concept that they are *bona fide* regulators of *Runx1* transcription.

## Discussion

4C analysis in HPC-7 cells generated a high-resolution connectivity map of the genomic region harbouring *Runx1*, and confirmed previously identified upstream connections from P1 to putative regulatory elements^18^. *Runx1* appears to be contained within a ~1 Mb chromatin domain, consistent with Hi-C analyses in other cell types^25,26^. We observed multiple connections both up- and downstream from all 4C-seq baits (*Runx1*-P1, *Runx1*-P2 and +24 enhancer). Significantly, there were many more upstream connections anchored by the active elements (*Runx1*-P1 and +24).

Surprisingly, the +24 enhancer forms many up- and downstream connections outside of the *Runx1* TAD. These comprise up to one-third of all connections formed and include other haematopoietic genes, such as *Tiam1* downstream, and *Erg* upstream. *Erg* and *Tiam1* dysregulation is associated with several tumor types, including AML and B- and T-cell lymphomas^27,28^. Strikingly, these genes are located over 2 Mb away from *Runx1*. These findings raise the interesting possibility that the +24 enhancer acts as a scaffold to recruit multiple promoters, enhancers and TFs over long distances in cis. Connections to +24 identified by our 4C-seq may point to the identity of some of these genes.

Our 4C-seq data identified multiple chromatin connections that intersect with some of the strongest indicators of upstream enhancers characterised by the binding of clusters of TFs and epigenetic modifications^18,20^. Eight out of nine of these DNA regions are able to drive haematopoietic expression in zebrafish, strongly suggesting an *in vivo* function. Although previous studies had identified transcriptional activity for a subset of these enhancers^15^, or an interaction with the P1 promoter for an overlapping subset^18^, no single prior study has shown that enhancers both interact with *Runx1* promoters (and +24), and have transcriptional activity. Table 1 provides an overview of findings from these previous studies together with additional evidence from our study that connects spatial proximity with function. Two enhancers, termed −59 and +110, were previously shown to drive *LacZ* expression in mice^15^; these are equivalent to the −58 and +110 enhancers identified here. Our data show in addition that these regions are in contact with P1 and the +24 enhancer (Table 1). While the −327 enhancer has similar characteristics as the other enhancers identified here, neither a previous study in mice^15^ nor our study identified any enhancer activity for it (Table 1). The −368, −354, −327, −321 and −303 enhancers were found to interact with P1 in a Capture Hi-C study in HPC-7 cells (Table 1). Our study confirms the presence of these interactions, and in addition, shows that these enhancers also interact with either the +24 enhancer or P2 (Table 1).

**Table 1:** Previous findings on enhancers and new findings from this study.

Enhancer location relative to P1 (kb)	Interactions identified by other studies	Previous functional validation	Findings from our study
−371	—	None	Interacts with +24 and P1. Enhancer activity in hematopoietic sites in 20–24 hpf zebrafish embryos.
−368	Interacts with P1 in HPC-7 cells (Capture Hi-C by Wilson *et al*.)^18^	None	Interacts with +24 and P1. Enhancer activity in hematopoietic sites in 20–24 hpf zebrafish embryos.
−354	Interacts with P1 in HPC-7 cells (Capture Hi-C by Wilson *et al*.)^18^	None	Interacts with +24 and P1. Enhancer activity in hematopoietic sites in 20–24 hpf zebrafish embryos.
−327	Interacts with P1 in HPC-7 cells (Capture Hi-C by Wilson *et al*.)^18^	No enhancer activity in mice (Schutte *et al*.)^15^	Interacts with +24 and P1. No enhancer activity in zebrafish.
−321	Interacts with P1 in HPC-7 cells (Capture Hi-C by Wilson *et al*.)^18^	Identified, but not tested for enhancer activity (Schutte *et al*.)^15^	Interacts with +24 and P1. Enhancer activity in hematopoietic sites in 20–24 hpf zebrafish embryos.
−303	Interacts with P1 in HPC-7 cells (Capture Hi-C by Wilson *et al*.)^18^	None	Interacts with +24 and P2. Enhancer activity in hematopoietic sites in 20–24 hpf zebrafish embryos and in keratinocytes from 20 hpf.
−58	—	Haematopoietic enhancer activity in E11.5 transgenic mice and luciferase enhancer activity in 416B cells (Schutte *et al*.)^15^	Interacts with +24 and P1. Enhancer activity in hematopoietic sites in 20–24 hpf zebrafish embryos.
−48	—	None	Interacts with +24 and P1. Enhancer activity in hematopoietic sites in 20–24 hpf zebrafish embryos.
+24	Interacts with P1 and P2 in human leukaemia cell lines (3 C by Markova *et al*.)^14^	Haematopoietic enhancer activity in mouse and zebrafish development and luciferase enhancer activity in 416B cells (Nottingham *et al*., 2009, Ng *et al*., 2010, Schutte *et al*.)^11,12,15^	Interacts with P1 and P2. Enhancer activity in hematopoietic sites in 20–24 hpf zebrafish embryos.
+110	—	Haematopoietic enhancer activity in E11.5 transgenic mice and luciferase enhancer activity in 416B cells (Schutte *et al*.)^15^	Interacts with P1. Enhancer activity in hematopoietic sites in 20–24 hpf zebrafish embryos.

The −303 enhancer interacts with P2 rather than P1 and, in addition to being active in haematopoietic sites, it drives expression in keratinocytes, indicating that it could act in a complex that keeps P2 silent; and/or that it regulates expression of *Runx1* in other tissues. Interestingly, *Runx1* is actively expressed in mouse keratinocytes where it is important for hair follicle development^29^. Neuronal cells also express *Runx1* ^30^, and there may be additional regulatory elements that control *Runx1* expression in a manner distinct from haematopoietic expression. In support of this idea, we previously determined that cohesin and CTCF influence *runx1* expression in haematopoietic, but not neuronal cells, in zebrafish^31,32^.

Cohesin and CTCF organise chromatin structure and when present in combination, they appear to negatively correlate with the HSPC TFs in HPC-7 cells^18^. However, we observed a coincidence of cohesin subunit Rad21 binding in the absence of CTCF with four out of nine identified enhancers, as well as the +24 enhancer. This suggests that CTCF-independent cohesin mediates a subset of enhancer-promoter looping in combination with TFs. This interpretation is consistent with previously identified CTCF-independent functions for cohesin in genome organization and transcription^33^. Importantly, cohesin mutations are prevalent in AML and other myeloid malignancies^34^, and are categorised together with *RUNX1* and spliceosome mutations in a genetic category that confers poor prognosis in AML^5^. Cohesin mutations led to increased chromatin accessibility of *Runx1* as measured by ATAC-seq.^35^, raising the possibility that spatiotemporal regulation of *Runx1* is cohesin-dependent in mouse and human, as was previously observed in zebrafish^31^.

 4C-seq in HPC-7 cells has provided new high resolution connectivity data that sheds light on the genomic organization of *Runx1*, an important haematopoietic transcription factor. These data confirm and extend previous analyses (Table 1), and furthermore, provide insight into the function of enhancers that have potential to regulate *Runx1* expression. The data presented here set the scene for functional analyses to precisely determine how *Runx1* is regulated, including CRISPR/Cas9-mediated interference with enhancer activity. They also provide a rationale for screening patients with myeloproliferative disorders for mutations in enhancer regions.

## Methods

### Cell culture

HPC-7 cells were maintained at a density of 1–10 × 10^5^ cells/mL in Iscove’s modified Dulbecco’s media (Gibco**®**) supplemented with 3.024 g/L sodium bicarbonate, 10% fetal bovine serum (Moregate, New Zealand), 10% stem cell factor conditioned media and 0.15 mM monothiolglycerol (Sigma-Aldrich) as previously described^36^. SCF-conditioned media was obtained from culturing BHK-MKL cells maintained in Dulbecco’s modified Eagle media (Sigma-Aldrich) supplemented with 3.5 g/L glucose, 3.7 g/L Sodium Bicarbonate and 10% FBS.

### 4C-seq library preparation

4C library preparation was performed as previously described^37^ with modifications. Three libraries were generated, two replicates (passage 8 and passage 10 cells) and one control (a 1:1 mix of both replicates for which the first ligation step was omitted). Cells were cross-linked in 2% formaldehyde, 5% FBS and 1x PBS for 10 minutes at room temperature while rotating. Formaldehyde was quenched with a final concentration of 125 mM glycine for 5 minutes on ice while inverting several times. Cell pellets were washed twice with ice-cold 1x PBS.

Nuclei were harvested by lysing the cell pellets in ice-cold lysis buffer (10 mM Tris pH 8.0, 10 mM NaCl, 0.2% NP-40, protease inhibitors) for 10 minutes on ice. Nuclei were then resuspended in 1.2x *Dpn*II restriction buffer (New England Biolabs) and 0.3% SDS and incubated for 1 hour at 37 °C while shaking. Triton X-100 was then added to a final concentration of 1.8% and the reaction was left at 37 °C while shaking for another hour. Chromatin was digested with 800 U of *Dpn*II overnight at 37 °C while shaking. *Dpn*II was inactivated by adding SDS to a final concentration of 1.3% and incubating at 65 °C for 20 minutes. Nuclei were diluted into a volume of 7 mL containing 1.01x T4 DNA ligase buffer (Life Technologies) and Triton-X100 at a final concentration of 1% and incubated at 37 °C for 1 hour. Ligations were carried out with 100 U of T4 ligase (Life Technologies) for 4.5 hours at 16 °C and 30 minutes at room temperature while shaking. For control libraries, ligase was omitted. Samples were proteinase K treated and reverse-crosslinked overnight at 65 °C. Samples were then treated with RNase A at 37 °C for 30 minutes. DNA was purified by phenol/chloroform extraction and ethanol precipitated.

A second digestion was performed with 25 U of *Bfa*I (New England Biolabs) for P1 and P2 baits or *Mse*I (New England Biolabs) for +24 baits in restriction buffer overnight at 37 °C while shaking. Restriction was inactivated by adding SDS to a final concentration of 1.3% and incubating at 65 °C for 20 minutes. A second ligation was performed in the same way as the first ligation, except that ligations were incubated overnight. DNA was purified with two phenol/chloroform extractions and one chloroform extraction followed by ethanol precipitation. DNA concentrations were measured using a Qubit® 3.0 Fluorometer (Life Technologies) and Qubit® double-stranded DNA (dsDNA) High Sensitivity Assay kit (Life Technologies).

For each bait, a total of 1 μg of DNA was amplified by PCR using Q5® High-Fidelity DNA Polymerase (New England Biolabs). Bait primer sequences are listed in Supplementary Table S3. PCR products were purified using the QIAquick PCR Purification kit (QIAGEN). DNA concentrations were measured using a Qubit® 3.0 Fluorometer and average fragment size by the 2100 Bioanalyser (Agilent Technologies) using a High Sensitivity DNA Kit (Agilent Technologies). Amplicons from the six different baits were mixed equally based on the concentration, average fragment size and ratio of demultiplexed 4C baits obtained from an initial MiSeq run. Libraries were prepared with Prep2Seq^TM^ DNA Library Prep Kit from Illumina^TM^ (Affymetrix) and TruSeq® adaptors (Illumina). Libraries were mixed equimolarly and sequenced as 125 bp paired-end reads on two Illumina^TM^ HiSeq 2500 lanes by New Zealand Genomics Limited.

### 4C-seq data analysis

4C-seq data was analysed in command-line and the R statistical environment^38^, and visualised using the University of California, Santa Cruz (UCSC) genome browser (http://genome.ucsc.edu/) with mouse assemblies mm9 or mm10^39,40,^ or with the R package ggplot2^41^. Baits were demultiplexed based on bait primer sequences up to and including the digestion site using a custom awk script, allowing 0 mismatches. Only read pairs that had the forward and reverse bait sequences in the correct orientation were selected. Adapter sequences, bait sequences up to but excluding the digestion site, and bases with a Phred quality score under 20 were then trimmed from the reads, using the fastq-multx, fastq-mcf and cleanadaptors v1.24 tools^42,43^. Quality of reads was assessed using FastQC (http://www.bioinformatics.babraham.ac.uk/projects/fastqc/)^44^.

Reads with a minimum length of 30 bp were mapped to the mm10 reference genome using Bowtie1^45^, allowing 0 mismatches. Mapped reads were assigned to *Dpn*II digestion fragments using fourSig^46^. The following reads were removed from the files: 1) self-ligated reads, 2) uncut reads (fragments adjacent to baits), and 3) reads at fragments that have at least 1 read in the control (non-ligated) library. The running mean was calculated from the sum of read counts from nine successive fragments, which was obtained using fourSig^46^, and was read per million normalised.

Significant interaction calling was performed using the R package fourSig with the following settings: window size of 3, 1000 iterations, fdr of 0.01, fdr.prob of 0.05 (which selects the top fifth percentile of interactions with a FDR of <0.01), and only included mappable fragments^46^. Significant interactions were called for two regions: 1) the whole of chr16, and 2) from chr16:92,250,000-93,635,000 (within the domain). Significant interactions in both replicates were overlapped using the bedIntersect tool from UCSC^47^.

In the fourSig package, significant interactions can be categorised into three categories: 1) interactions that are significant after the reads from the fragment with the highest read count is removed, 2) interactions that are significant when the fragment with the highest read count is averaged to the read counts of the neighbouring fragments, and 3) interactions that are significant only when all fragment read counts are included^46^. For this study, only category 1 and 2 interactions that overlap between both replicates were included, as they are more likely to represent true interactions (because they span multiple fragments), and were previously shown to be more reproducible between replicates than single-fragment interactions^46^. Furthermore, we distinguished category 1 interactions that overlap between both replicates from other category 1 and 2 interactions by colouring them red and orange, respectively, to visualise the most significant interactions. For conversion from assembly mm10 to mm9, the liftOver tool from UCSC was used (http://genome.ucsc.edu/)^47^. Gene annotations used in Figures are UCSC reference genes.

### Zebrafish enhancer assay

*Runx1* regulatory regions were amplified from HPC-7 gDNA or from I-SceI-zhsp70 plasmid containing the −368, +24 and +110 sequences^11^ (primer sequences are in Supplementary Table S3), and cloned into the zebrafish enhancer detection vector^48^. Plasmid purifications were performed with the NucleoSpin® Plasmid or NucleoBond® Xtra Midi prep kits (Machery-Nagel). Primers amplified the TF binding peak +/− 200 bp, except for −321 which (due to a repetitive region) did not have a 200-bp extension on the 3′ end. The −368, +24 and +110 fragments are 471, 529 and 579 bp, respectively^11^. A mixture of 30 pg vector DNA and 120 pg Tol2 transposase mRNA^49^ was injected into 1-cell zebrafish embryos. Embryos were imaged at 20–24 or ~48 hpf using a Leica M205FA stereomicroscope with a DFC490 camera and LAS software (Leica Microsystems), images were processed using Adobe Photoshop. Zebrafish were maintained as described previously^50^ and zebrafish handling and procedures were carried out in accordance with the Otago Zebrafish Facility Standard Operating Procedures. The University of Otago Animal Ethics Committee approved all zebrafish research under approval AEC 48/11.

### Identification of conserved non-coding elements

Mouse conserved non-coding elements (mCNEs) were identified as described previously^11^.

### ChIP-seq, Capture Hi-C and DNase I hypersensitivity data

Occupancy of the transcription factors Erg, Fli1, Scl, Runx1, Gata2, E2A, Ldb1, Lyl1, Lmo2, Gfi1b, Meis1, Myb, phospho-Stat1, Pu.1, Stat3, Eto2, Cebp-α, Cebp-β, Elf1, Nfe2, p53, cMyc, Egr1, E2f4, cFos, Mac and Jun; Rad21 and CTCF; H3K27ac and H3K4me3; DNase I hypersensitivity sites; and Capture Hi-C data in HPC-7 cells was obtained from previously published data^18–20^. Rad21, Smc3 and CTCF chromatin immunoprecipitation sequencing (ChIP-seq) data in MEL and CH12 cells were obtained from ENCODE^51^.

### Availability of data

The 4C-seq dataset is accessible through GEO Series accession number GSE86994 (https://www.ncbi.nlm.nih.gov/geo/query/acc.cgi?acc = GSE86994).

## Electronic supplementary material

Supplementary Information
