# Supplementary material for: A DNA Contact Map for the Mouse Runx1 Gene Identifies Novel Haematopoietic Enhancers
Source: Sci Rep. 2017 Oct 17;7:13347. doi: 10.1038/s41598-017-13748-8 (PMC5645309; doi:10.1038/s41598-017-13748-8)
Supplement: Supplementary file 1 — Supplementary Information [file 41598_2017_13748_MOESM1_ESM.pdf]

Supplementary Information

A DNA Contact Map for the Mouse *Runx1* Gene Identifies Novel Haematopoietic Enhancers

Judith Marsman, Amarni Thomas, Motomi Osato, Justin M. O'Sullivan and Julia A. Horsfield

Supplementary Figures and Legends

a

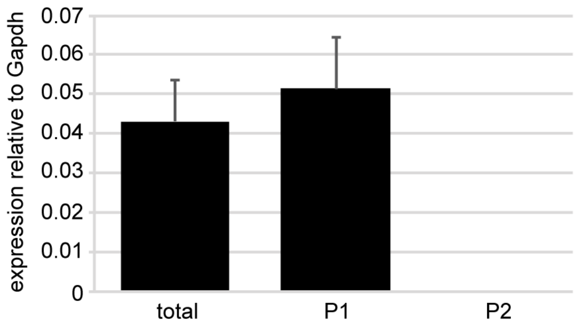

b

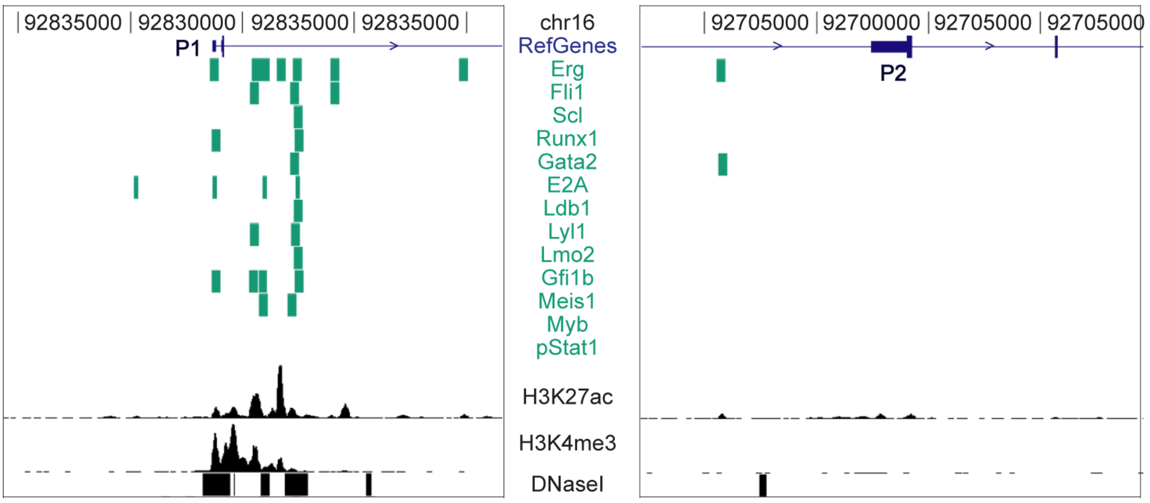

Supplementary Figure S1. Activity of the *Runx1* P1 and P2 promoters in HPC-7 cells. (**a**) Transcript levels of *Runx1*, *Runx1*-P1 (*Runx1c*) and *Runx1*-P2 (*Runx1a* and *Runx1b*) in HPC-7 cells were measured by quantitative RT-PCR and are expressed relative to *Gapdh* (mean ± SEM, n = 8 replicates). (**b**) Binding of haematopoietic progenitor transcription factors (green), H3K27ac, H3K4me3 and DNaseI hypersensitivity sites in HPC-7 cells[1-3] at the *Runx1* P1 and P2 promoters are shown (mm9). Haematopoietic transcription factor binding peaks shown are of Erg, Fli1, Scl, Runx1, Gata2, E2A, Ldb1, Lyl1, Lmo2, Gfi1b, Meis1, Myb and pStat1.

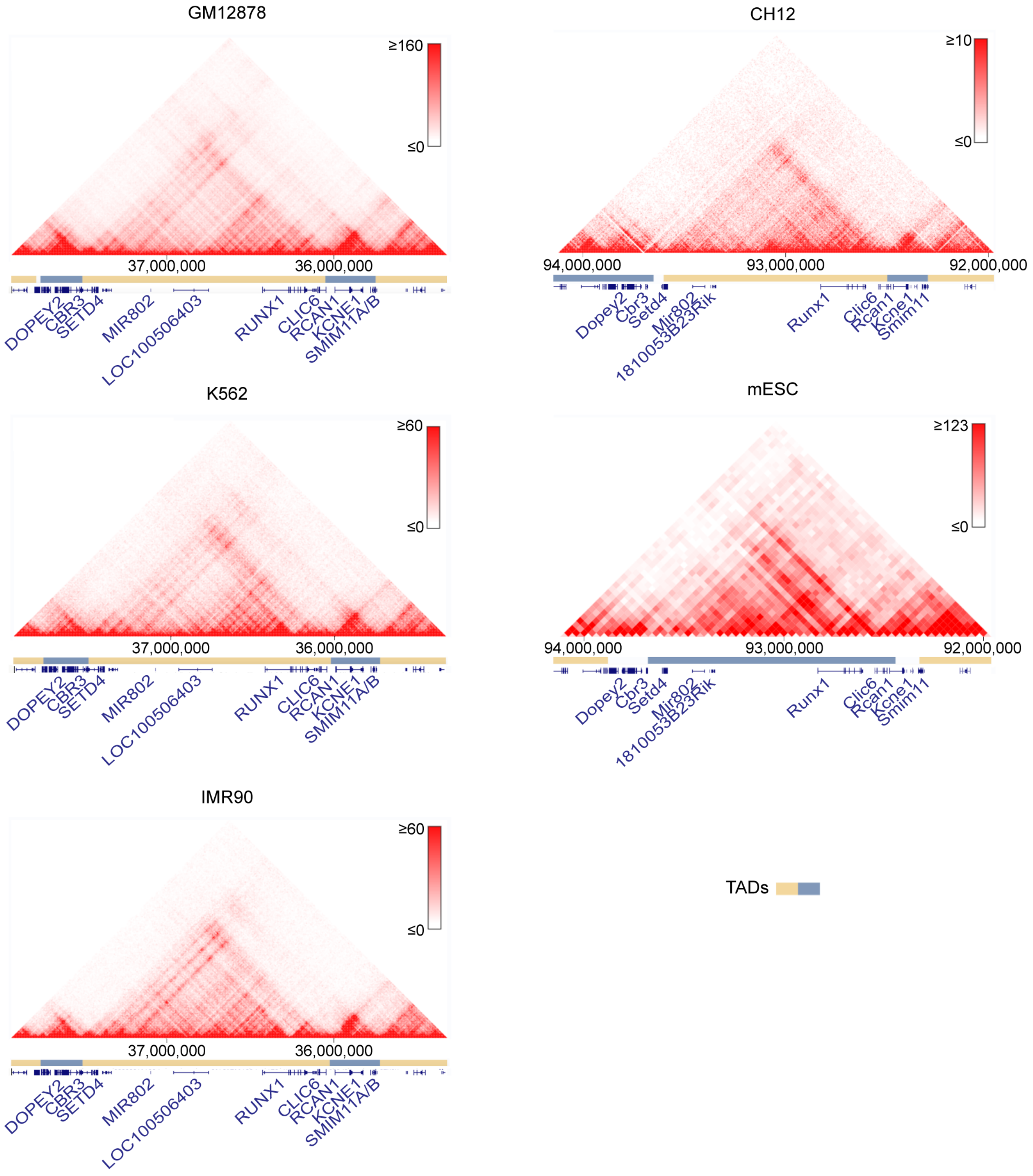

Supplementary Figure S2. Hi-C contact maps in GM12878, K562, IMR90, CH12 and mESC cells. Hi-C contact maps in a region surrounding *Runx1* were obtained using the Hi-C genome browser (http://www.3dgenome.org)[4,5,6]. TADs, calculated with the directional bias method[4], are indicated by alternating orange and blue bars. UCSC reference genes (using mouse assembly mm9 and human assembly hg19) are annotated.

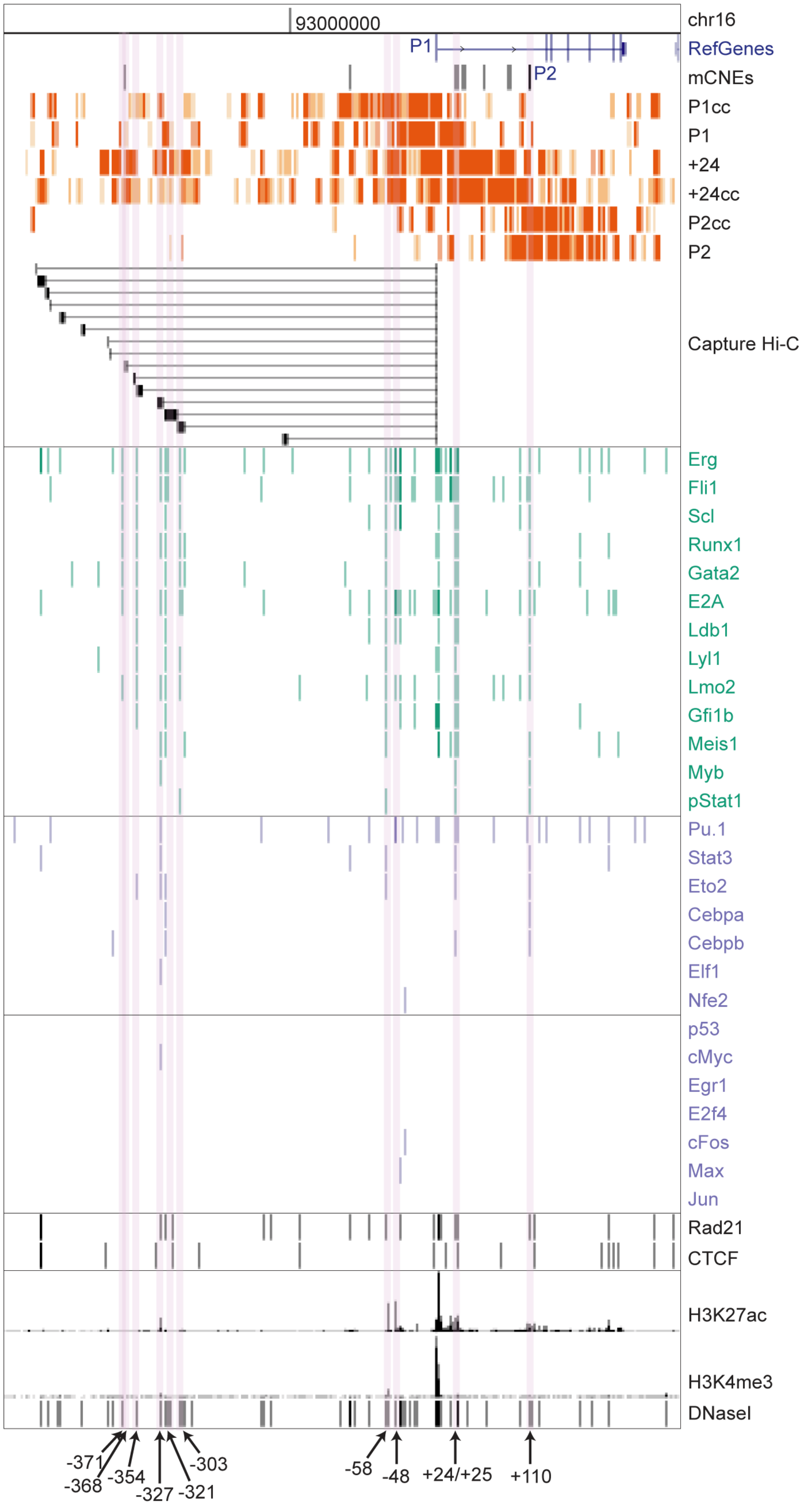

93000000
chr16
P1
RefGenes
P2
mCNEs
P1cc
P1
+24
+24cc
P2cc
P2
Capture Hi-C
Erg
Fli1
Scl
Runx1
Gata2
E2A
Ldb1
Lyl1
Lmo2
Gfi1b
Meis1
Myb
pStat1
Pu.1
Stat3
Eto2
Cebpa
Cebpb
Elf1
Nfe2
p53
cMyc
Egr1
E2f4
cFos
Max
Jun
Rad21
CTCF
H3K27ac
H3K4me3
DNaseI
-371
-368
-354
-327
-321
-303
-58
-48
+24/+25
+110

Supplementary Figure S3. Comparison of *Runx1* 4C-seq with Capture Hi-C data, transcription factor binding sites and epigenetic modifications in HPC-7 cells. Significant interactions (with the most highly significant interactions in red and other significant interaction in orange) within the domain for each bait are shown together with reference genes (assembly mm9), mouse conserved non-coding elements (mCNEs)[7], transcription factor binding sites involved in haematopoietic progenitor cell development (green), other myeloid and general transcription factor binding sites (purple), Rad21 and CTCF binding sites (black), H3K27ac, H3K4me3, DNaseI hypersensitivity sites and Capture Hi-C data of the P1 promoter in HPC-7 cells[1-3]. Transcription factor binding peaks involved in haematopoietic progenitor cell development are of Erg, Fli1, Scl, Runx1, Gata2, E2A, Ldb1, Lyl1, Lmo2, Gfi1b, Meis1, Myb and pStat1, other myeloid transcription factor binding sites are of Pu.1, Stat3, Eto2, Cebpa, Cebpb, Elf1 and Nfe2, and general transcription factor binding sites are of p53, cMyc, Egr1, E2f4, cFos, Mac and Jun. Locations of putative haematopoietic enhancers are indicated by arrows, and named according to their distance from the P1 transcriptional start site. Related to Fig. 3.

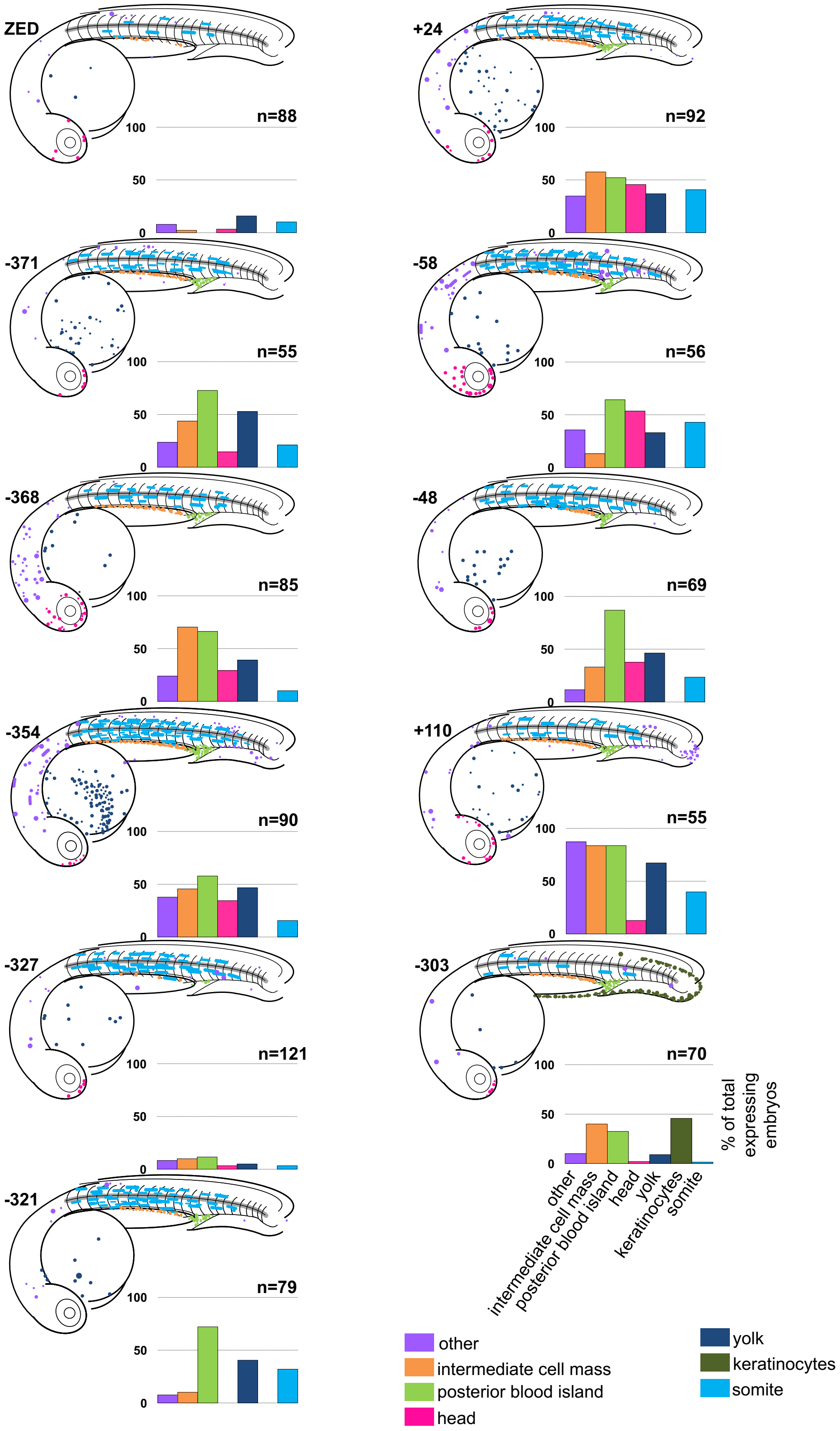

Supplementary Figure S4. Quantitative summary of GFP expression patterns generated by putative mouse enhancers in zebrafish. For each enhancer construct (+110, -48, -58, -303, -321, -327, -354, -368, -371) and the negative (ZED) and positive (+24) controls, GFP expression data in different tissues is shown. Diagrams represent pooled expression from at least 5 photographed embryos per putative enhancer (20-24 hpf),

displayed on illustrations of a 24 hpf zebrafish embryo. Categories of expression zones are colour-coded: key is at the bottom of the Figure. Bar graphs display the percentage of the total number of GFP-expressing embryos that show expression in each tissue category for each putative enhancer. The total number of expressing embryos analysed per construct is displayed in the top right corner of each graph. Related to Figure 5.

Supplementary Tables

Supplementary Table S1. Genomic characteristics of 4C-seq-identified enhancers.

**Enhancer location relative to P1 (kb)**	**Interaction with 4C-seq bait**	**Binding of haematopoietic progenitor cell TFs in HPC-7 cells**	**Binding of other TFs in HPC-7 cells**	**Rad21 binding, no CTCF**	**DNaseI site**	**Previously identified to be in a CNE** (Ng *et al.*, 2010)[7]
-371	+24, P1 (within 2 kb)	Erg, Fli1, Scl, Runx1, Gata2, E2A, Lmo2			✓	
-368	+24, P1					✓
-354	+24, P1	Erg, Fli1, Scl, Runx1, Gata2, E2A, Ldb1, Lyl1, Lmo2, Gfi1b	Eto2		✓	
-327	+24, P1	Erg, Fli1, Runx1, E2A, Lmo2, Meis1, Myb	Pu. 1, Stat3, Eto2, Elf1, cMyc	✓	✓	
-321	+24, P1	Erg, Fli1, Scl, Runx1, Gata2, E2A, Ldb1, Lyl1, Lmo2, Gfi1b, Meis1	Eto2, Cebpa, Cebpb	✓	✓	
-303	+24, P2 (within 2 kb)	Erg, Fli1, Scl, Runx1, Gata2, E2A, Lyl1, Lmo2, pStat1			✓	
-58	+24, P1	Erg, Fli1, Scl, Runx1, Gata2, E2A, Ldb1, Lyl1, Lmo2, Gfi1b, Meis1, pStat1	Stat3, Eto2	✓	✓	
-48	+24, P1 (within 2 kb)	Erg, Fli1, Scl, E2A, Ldb1, Lmo2	Pu. 1		✓	
+24	P1, P2 (within 2 kb)	Erg, Fli1, Scl, Runx1, Gata2, E2A, Ldb1, Lyl1, Lmo2, Gfi1b, Meis1, Myb, pStat1	Pu. 1, Stat3, Eto2, Cebpb	✓	✓	✓
+110	+24, P1	Erg, Fli1, Scl, Runx1, Gata2, E2A, Ldb1, Lyl1, Lmo2, Meis1, Myb, pStat1	Pu. 1, Stat3, Eto2, Cebpa, Cebpb	✓	✓	✓

Supplementary Table S2. Transcription factor binding motif prediction at *Runx1* enhancers.

**Transcription factor motif class**	**-371**	**-368**	**-354**	**-327**	**-321**	**-303**	**-58**	**-48**	**+24**	**+110**
Homeo domain	✓	✓	✓	✓	✓	✓	✓	✓	✓	✓
C2H2 zinc finger	✓	✓	✓	✓	✓	✓	✓	✓	✓	✓
Basic helix-loop-helix	✓	✓	✓	✓		✓	✓	✓	✓	✓
Tryptophan cluster	✓	✓	✓	✓			✓	✓	✓	✓
GATA-type zinc fingers	✓		✓		✓	✓	✓	✓	✓	✓
SMAD/NF-1 DNA-binding domain factors	✓	✓		✓	✓	✓	✓	✓		✓
ARID domain	✓	✓		✓	✓	✓		✓		✓
Fork head/winged helix		✓	✓	✓	✓	✓				✓
High-mobility group domain			✓		✓		✓	✓		✓
Runt domain				✓	✓				✓	
Nuclear receptors with C4 zinc fingers						✓		✓		✓
Paired box		✓	✓			✓				
STAT domain	✓	✓		✓						
Basic leucine zipper	✓				✓		✓			
Rel homology region		✓	✓							
Basic helix-span-helix factors										✓
TEA domain factors			✓							
T-Box factors									✓	

Supplementary Table S3. Primer sequences.

4C primers

Primer name	*Sequence (5′-3′)*
4Cm_DpnII+BfaI_P1cc_F	TGCAGCTACAGGCTTGGAT
4Cm_DpnII+BfaI_P1cc_R	CAGCAGTTAAAGCCGCTCTC
4Cm_DpnII+BfaI_P1_F	GAGAGGCAGCTCACAGGTAT
4Cm_DpnII+BfaI_P1_R	GGATTTGGTGGCTTTCAGAT
4Cm_DpnII+MseI_+24_F	GGGACCATTGCTTTCCATAA
4Cm_DpnII+MseI_+24_R	TCAGGAGCGTGTCAGGAAG
4Cm_DpnII+MseI_+24cc_F	ATGTCATCGTGAAACCTGCT
4Cm_DpnII+MseI_+24cc_R	GCAAAGGTGAACTTGAAACTCT
4Cm_DpnII+BfaI_P2cc_F	CCCCATGCTTTCAACTTTT
4Cm_DpnII+BfaI_P2cc_R	TTGGGGTGGCTTCTCTTG
4Cm_DpnII+BfaI_P2_F	CCCTTTGCGGCTCTTTCTA
4Cm_DpnII+BfaI_P2_R	CACACGCTGCCATGTTTG

Primers used for cloning of putative enhancers

Primer name	*Sequence (5′-3′)*
-371F	AATCCCCAGGATGCCTTTAG
-371R	TCAGACCTCCCATTCCTGAC
-354F	AAGAGTTCCCTGCCTGGATT
-354R	TATAGCACAATGGCCTGCAC
-327F	GACAGACTCTGGGGATGGAA
-327R	GCCTGGTATTTGGAGTCTGG
-321F	TCCATCTCTTGTGTGTTGCAG
-321R	TCTCCCAGGCTACCTCTTGA
-303F	CCACTTTGGGAGGTGTGAAT
-303R	CATGGAGGGAGGGTGTGTAG
-58F	GGACCCTTGGTAACTCGACA
-58R	TTTGCCAAGGAAAGCACTTC
-48F	TGAAAGGTGATTGGTCCTCA
-48R	CCATGATCTCTCCACCATGA
I-SceI-zhsp70-F	AAAGGGAACAAAAGCTGGAG
I-SceI-zhsp70-R	AGTCGCTTCTCTTCGGTTGA

Primers used for quantitative PCR

Primer name	*Sequence (5′-3′)*
Runx1-P1-F	AGCCTGGCAGTGTCAGAAGT
Runx1-P1-R	CTTTCGAAAACGCACCTCTC
Runx1-P2-F	GTGATGCGTATCCCCGTAG
Runx1-P2-R	ATGACGGTGACCAGAGTGC
Runx1-F	GCCATGAAGAACCAGGTAGC
Runx1-R	GACGGTGATGGTCAGAGTGA
Gapdh-F	GGTGCTGAGTATGTCGTGGA
Gapdh-R	CGGAGATGATGACCCTTTTG

Supplementary Methods

Quantitative reverse transcription PCR
Total RNA from HPC-7 cells was extracted using the Nucleospin® RNA II Kit (Machery-Nagel) and 500 ng of extracted RNA was used to synthesise cDNA (qScript™ cDNA SuperMix, Quanta Biosciences™). Takara Bio SYBR® Premix *Ex Taq™* (Tli RNase H Plus; Clontech) was used to amplify cDNA in 10-µL reactions in a 96-well plate (LightCycler® 480 Multiwell Plate 96, Roche Diagnostics) on the LightCycler 480 (Roche Diagnostics). Cp values were determined by the 2nd derivative method using Roche LightCycler® 480 software. PCR primers are listed in Supplementary Table S3.

Hi-C data
Previously described Hi-C data in human GM12878, K562 and IMR90 cells, and mouse CH12 and embryonic stem cells was obtained using the Hi-C genome browser (http://www.3dgenome.org)[4,5,6].

***In silico* transcription factor binding motif prediction**
In silico transcription factor binding motifs for the -371, -368, -354, -327, -321, -303, -58, -48, +24 and +110 enhancers were predicted using JASPAR Core Vertebrata motifs for human and mouse[8]. Motifs with a score of >95% were selected. Enhancer DNA sequences for motif analysis were selected as the enhancer centre (defined as the middle of the most up- and downstream TF binding site) +/- 300 bp up- and downstream.

Supplementary References

1. Calero-Nieto, F.J. *et al.* Key regulators control distinct transcriptional programmes in blood progenitor and mast cells. *EMBO J* **33**, 1212-26 (2014).
2. Wilson, N.K. *et al.* Combinatorial transcriptional control in blood stem/progenitor cells: genome-wide analysis of ten major transcriptional regulators. *Cell Stem Cell* **7**, 532-44 (2010).
3. Wilson, N.K. *et al.* Integrated genome-scale analysis of the transcriptional regulatory landscape in a blood stem/progenitor cell model. *Blood* **127**, e12-e23 (2016).
4. Dixon, J.R. *et al.* Topological domains in mammalian genomes identified by analysis of chromatin interactions. *Nature* **485**, 376-80 (2012).
5. Rao, S.S. *et al.* A 3D map of the human genome at kilobase resolution reveals principles of chromatin looping. *Cell* **159**, 1665-80 (2014).
6. Wang, Y. *et al*. The 3D Genome Browser: a web-based browser for visualizing 3D genome organization and long-range chromatin interactions. *BioRxiv* (2017).
7. Ng, C.E. *et al.* A Runx1 intronic enhancer marks hemogenic endothelial cells and hematopoietic stem cells. *Stem Cells* **28**, 1869-81 (2010).
8. Mathelier, A. *et al.* JASPAR 2016: a major expansion and update of the open-access database of transcription factor binding profiles. *Nucleic Acids Res* **44**, D110-5 (2016).
